# (2-Carbamoylethyl-κ^2^ *C* ^1^,*O*)triiodidotin(IV)

**DOI:** 10.1107/S1600536811041778

**Published:** 2011-10-12

**Authors:** Geraldo M. de Lima, Edward R. T. Tiekink, James L. Wardell, Solange M. S. V. Wardell

**Affiliations:** a Departamento de Química, Instituto de Cie^ncias Exatas, Universidade Federal de Minas Gerais, Avenida Anto^nio Carlos, 6627 Pampulha, 31270-901 Belo Horizonte, MG, Brazil; b Department of Chemistry, University of Malaya, 50603 Kuala Lumpur, Malaysia; c Centro de Desenvolvimento Tecnológico em Saúde (CDTS), Fundação Oswaldo Cruz (FIOCRUZ), Casa Amarela, Campus de Manguinhos, Av. Brasil 4365, 21040-900 Rio de Janeiro, RJ, Brazil; d CHEMSOL, 1 Harcourt Road, Aberdeen AB15 5NY, Scotland

## Abstract

Two independent but virtually identical mol­ecules comprise the asymmetric unit of the title compound, [Sn(C_3_H_6_NO)I_3_]. The CI_3_O coordination geometry around the Sn^IV^ atom is defined by a chelating carbamoylethyl ligand (*C* ^1^,*O*-bidentate) and three I atoms, and is based on a distorted trigonal bipyramid with the carbonyl O atom occupying a position *trans* to one of the I atoms which forms the longer of the Sn—I bonds. The independent mol­ecules are linked *via* N—H⋯O hydrogen bonds, which leads to the formation on an eight-membered amide {⋯HNCO}_2_ synthon. N—H⋯I hydrogen-bonding inter­actions are also present between neighbouring mol­ecules.

## Related literature

For background to and for related Sn[OCH(NH_2_)CH_2_CH_2_]Cl_3_ *L* structures (*L* = amide), see: Howie *et al.* (2011*a* ▶,*b* ▶); Wardell *et al.* (2010 ▶); Tiekink *et al.* (2006 ▶). For additional geometric analysis, see: Addison *et al.* (1984 ▶); Spek (2009 ▶).
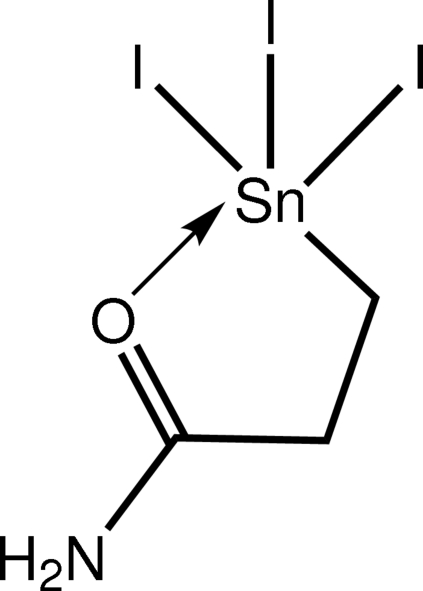


## Experimental

### 

#### Crystal data

[Sn(C_3_H_6_NO)I_3_]
*M* *_r_* = 571.48
Triclinic, 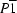

*a* = 7.8530 (1) Å
*b* = 10.6264 (1) Å
*c* = 14.1250 (2) Å
α = 98.801 (1)°
β = 105.523 (1)°
γ = 102.383 (1)°
*V* = 1081.22 (2) Å^3^
*Z* = 4
Mo *K*α radiation
μ = 10.87 mm^−1^
*T* = 120 K
0.20 × 0.20 × 0.02 mm

#### Data collection

Bruker–Nonius APEXII CCD diffractometer
Absorption correction: multi-scan (*SADABS*; Sheldrick, 2007 ▶) *T*_min_ = 0.379, *T*_max_ = 1.000
14061 measured reflections
4414 independent reflections
4342 reflections with *I* > 2σ(*I*)
*R*_int_ = 0.039

#### Refinement

*R*[*F* ^2^ > 2σ(*F* ^2^)] = 0.027
*wR*(*F* ^2^) = 0.079
*S* = 1.12
4414 reflections
163 parameters
H-atom parameters constrained
Δρ_max_ = 1.37 e Å^−3^
Δρ_min_ = −1.46 e Å^−3^

### 

Data collection: *COLLECT* (Hooft, 1998 ▶); cell refinement: *DENZO* (Otwinowski & Minor, 1997 ▶) and *COLLECT*; data reduction: *DENZO* and *COLLECT*; program(s) used to solve structure: *SHELXS97* (Sheldrick, 2008 ▶); program(s) used to refine structure: *SHELXL97* (Sheldrick, 2008 ▶); molecular graphics: *ORTEP-3* (Farrugia, 1997 ▶) and *DIAMOND* (Brandenburg, 2006 ▶); software used to prepare material for publication: *publCIF* (Westrip, 2010 ▶).

## Supplementary Material

Crystal structure: contains datablock(s) global, I. DOI: 10.1107/S1600536811041778/wm2541sup1.cif

Structure factors: contains datablock(s) I. DOI: 10.1107/S1600536811041778/wm2541Isup2.hkl

Additional supplementary materials:  crystallographic information; 3D view; checkCIF report

## Figures and Tables

**Table 1:** Selected bond lengths (Å)

Sn1—C1	2.146 (5)
Sn1—O1	2.347 (3)
Sn1—I1	2.6953 (4)
Sn1—I2	2.7796 (4)
Sn1—I3	2.6904 (4)
Sn2—C4	2.147 (5)
Sn2—O2	2.330 (3)
Sn2—I4	2.6987 (4)
Sn2—I5	2.6880 (4)
Sn2—I6	2.8060 (4)

**Table 2:** Hydrogen-bond geometry (Å, °)

*D*—H⋯*A*	*D*—H	H⋯*A*	*D*⋯*A*	*D*—H⋯*A*
N1—H1n⋯O2	0.88	2.29	3.085 (7)	150
N2—H3n⋯O1	0.88	2.26	3.018 (7)	145
N2—H4n⋯I1	0.88	3.06	3.784 (6)	141

**Fig. 1.**
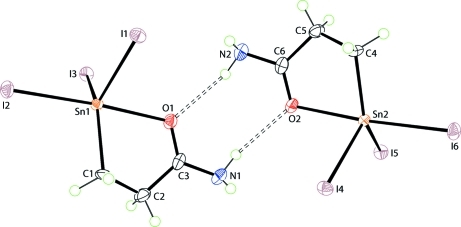
The molecular structure of the two independent molecules comprising the asymmetric unit in (I) showing the atom-labelling scheme and displacement ellipsoids at the 50% probability level. The N—H···O hydrogen bonds are shown as dashed lines.

## supplementary crystallographic information

### Comment

The title compound, (I), was studied as a continuation of structural investigations of 2-amidoethyl compounds of stannanes(IV) (Tiekink *et al.*, 2006; Wardell *et al.*, 2010; Howie *et al.*, 2011**a*,b*, and references therein).

Two independent molecules comprise the asymmetric unit of (I), (Fig. 1). The two molecules are virtually identical with the r.m.s. deviations for distances and angles being 0.0132 Å and 3.291°, respectively (Spek, 2009). The greatest difference in equivalent bond lengths is found in the Sn1—I2 and Sn2—I6 bonds (Table 1). The Sn^IV^ atom in each molecule is chelated by the amidoethyl ligand and additionally coordinated by three I atoms. Each of the five-membered chelate rings is twisted, with the twist occurring about the CH_2_—CH_2_ bond in each case. The resulting CI_3_O donor set defines a coordination geometry intermediate between square-pyramidal and trigonal-bipyramidal, with a leaning towards the latter description. This is quantified by the value of τ = 0.80 [Sn1] which compares to the τ values of 0.0 and 1.0 for ideal square-pyramidal and trigonal-bipyramidal geometries, respectively (Addison *et al.*, 1984). The τ value for the Sn2 atom is 0.72. The disparity in the Sn—I bond lengths (Table 1), shows that the I atoms in the axial positions, each of which is *trans* to an O atom, form longer bonds than the I atoms occupying equatorial positions.

It is of interest that while (H_2_NCOCH_2_CH_2_-*C*,*O*)SnCl_3_ readily forms six-coordinate complexes, [(H_2_NCOCH_2_CH_2_-*C*,*O*)SnCl_3_.*L*] with oxygen ligands, *L*, *e.g. L* = amide, as illustrated by the isolation of [(H_2_NCOCH_2_CH_2_-*C*,*O*)(EtCONH_2_-*O*)SnCl_3_] from reaction mixtures containing SnCl_2_, HCl and H_2_C═CHCONH_2_ in Et_2_O (Howie *et al.*, 2011*b*), the triiodido analogue is reluctant to form similar complexes. This is a consequence of the reduced Lewis acidity of the tin atom in iodidostannanes compared to chloridostannanes.

The two molecules comprising the asymmetric unit are linked *via* N—H···O hydrogen bonds, leading to the formation of an eight-membered {···HNCO}_2_ synthon (Fig. 1, Table 2). The other H atom on each N forms an interaction with an I atom of the other molecule, in the the case of the N1—H2n atom, this distance is long at 3.14 Å.

### Experimental

A solution of the complex, (H_2_NCOCH_2_CH_2_—C,O)(EtCONH_2_-O)SnCl_3_ (0.74 g, 2 mmol), isolated from a reaction mixture containing SnCl_2_, HCl and H_2_C═CHCONH_2_ in Et_2_O (Howie *et al.*, 2011*b*), and sodium iodide (10 mmol) in acetone (30 ml) was refluxed for 3 h, filtered to remove sodium chloride and rotary evaporated. The residue was extracted into chloroform (30 ml), the organic solution was rotary evaporated and the resulting residue was recrystallized from ethanol to give the title compound, melting point 461–463 K. IR: ν(C═O) 1660, 1581 cm^-1^.

### Refinement

The C-bound H atoms were geometrically placed (N—H = 0.88 Å and C—H = 0.99 Å) and refined as riding with *U*_iso_(H) = 1.2*U*_eq_(N, C). The maximum and minimum residual electron density peaks of 1.37 and 1.46 e^-^ Å^-3^, respectively, are located 1.34 Å and 0.85 Å from the I5 and I3 atoms, respectively.

### Crystal data

**Table:**

[Sn(C_3_H_6_NO)I_3_]	*Z* = 4
*M**_r_* = 571.48	*F*(000) = 992
Triclinic, *P*1	*D*_x_ = 3.511 Mg m^−^^3^
Hall symbol: -P 1	Mo *K*α radiation, λ = 0.71073 Å
*a* = 7.8530 (1) Å	Cell parameters from 4411 reflections
*b* = 10.6264 (1) Å	θ = 2.9–27.5°
*c* = 14.1250 (2) Å	µ = 10.87 mm^−^^1^
α = 98.801 (1)°	*T* = 120 K
β = 105.523 (1)°	Plate, yellow
γ = 102.383 (1)°	0.20 × 0.20 × 0.02 mm
*V* = 1081.22 (2) Å^3^	

### Data collection

**Table:**

Bruker–Nonius APEXII CCD diffractometer	4414 independent reflections
Radiation source: Bruker-Nonius FR591 rotating anode	4342 reflections with *I* > 2σ(*I*)
10cm confocal mirrors	*R*_int_ = 0.039
Detector resolution: 9.091 pixels mm^-1^	θ_max_ = 26.5°, θ_min_ = 3.0°
φ and ω scans	*h* = −9→9
Absorption correction: multi-scan (*SADABS*; Sheldrick, 2007)	*k* = −13→13
*T*_min_ = 0.379, *T*_max_ = 1.000	*l* = −17→17
14061 measured reflections	

### Refinement

**Table:**

Refinement on *F*^2^	Primary atom site location: structure-invariant direct methods
Least-squares matrix: full	Secondary atom site location: difference Fourier map
*R*[*F*^2^ > 2σ(*F*^2^)] = 0.027	Hydrogen site location: inferred from neighbouring sites
*wR*(*F*^2^) = 0.079	H-atom parameters constrained
*S* = 1.12	*w* = 1/[σ^2^(*F*_o_^2^) + (0.0384*P*)^2^ + 3.8857*P*] where *P* = (*F*_o_^2^ + 2*F*_c_^2^)/3
4414 reflections	(Δ/σ)_max_ = 0.001
163 parameters	Δρ_max_ = 1.37 e Å^−^^3^
0 restraints	Δρ_min_ = −1.46 e Å^−^^3^

### Special details

**Table:**

Geometry. All s.u.'s (except the s.u. in the dihedral angle between two l.s. planes) are estimated using the full covariance matrix. The cell s.u.'s are taken into account individually in the estimation of s.u.'s in distances, angles and torsion angles; correlations between s.u.'s in cell parameters are only used when they are defined by crystal symmetry. An approximate (isotropic) treatment of cell s.u.'s is used for estimating s.u.'s involving l.s. planes.
Refinement. Refinement of *F*^2^ against ALL reflections. The weighted *R*-factor *wR* and goodness of fit *S* are based on *F*^2^, conventional *R*-factors *R* are based on *F*, with *F* set to zero for negative *F*^2^. The threshold expression of *F*^2^ > 2σ(*F*^2^) is used only for calculating *R*-factors(gt) *etc*. and is not relevant to the choice of reflections for refinement. *R*-factors based on *F*^2^ are statistically about twice as large as those based on *F*, and *R*- factors based on ALL data will be even larger.

### Fractional atomic coordinates and isotropic or equivalent isotropic displacement parameters (Å^2^)

**Table:**

	*x*	*y*	*z*	*U*_iso_*/*U*_eq_	
Sn1	0.84744 (4)	0.81052 (3)	0.07941 (2)	0.01343 (9)	
I1	0.79233 (5)	0.56511 (3)	0.11465 (2)	0.02019 (10)	
I2	1.13298 (5)	0.80096 (3)	−0.00043 (3)	0.02164 (10)	
I3	0.58439 (4)	0.79311 (3)	−0.09302 (2)	0.01808 (9)	
O1	0.6302 (5)	0.8274 (4)	0.1636 (3)	0.0196 (7)	
N1	0.5852 (8)	0.9523 (5)	0.2928 (4)	0.0324 (12)	
H1N	0.4849	0.8870	0.2762	0.039*	
H2N	0.6529	0.9568	0.3546	0.039*	
C1	0.9830 (7)	0.9848 (5)	0.1974 (4)	0.0190 (10)	
H1A	1.0707	0.9634	0.2533	0.023*	
H1B	1.0533	1.0528	0.1714	0.023*	
C2	0.8454 (9)	1.0397 (5)	0.2370 (4)	0.0266 (12)	
H2A	0.9052	1.0874	0.3083	0.032*	
H2B	0.8057	1.1037	0.1978	0.032*	
C3	0.6791 (7)	0.9313 (5)	0.2296 (4)	0.0198 (10)	
Sn2	0.27996 (4)	0.67003 (3)	0.46025 (2)	0.01266 (9)	
I4	0.61233 (4)	0.84602 (3)	0.54333 (3)	0.01904 (10)	
I5	0.02318 (4)	0.80393 (3)	0.43118 (2)	0.01823 (9)	
I6	0.25104 (5)	0.60910 (3)	0.64270 (2)	0.02072 (10)	
O2	0.3235 (5)	0.6994 (4)	0.3074 (3)	0.0208 (8)	
N2	0.3498 (7)	0.5858 (5)	0.1668 (4)	0.0286 (11)	
H3N	0.3856	0.6666	0.1585	0.034*	
H4N	0.4401	0.5480	0.1716	0.034*	
C4	0.2650 (7)	0.4737 (5)	0.3869 (4)	0.0171 (9)	
H4A	0.3835	0.4533	0.4140	0.021*	
H4B	0.1684	0.4090	0.4005	0.021*	
C5	0.2215 (7)	0.4620 (5)	0.2734 (4)	0.0213 (11)	
H5A	0.2708	0.3921	0.2449	0.026*	
H5B	0.0868	0.4358	0.2414	0.026*	
C6	0.3029 (7)	0.5905 (5)	0.2496 (4)	0.0188 (10)	

### Atomic displacement parameters (Å^2^)

**Table:**

	*U*^11^	*U*^22^	*U*^33^	*U*^12^	*U*^13^	*U*^23^
Sn1	0.01254 (17)	0.01471 (17)	0.01161 (16)	0.00316 (13)	0.00254 (13)	0.00162 (12)
I1	0.02194 (19)	0.01739 (17)	0.02113 (18)	0.00547 (14)	0.00490 (14)	0.00694 (13)
I2	0.01880 (18)	0.02449 (18)	0.02683 (19)	0.00877 (14)	0.01201 (14)	0.00795 (14)
I3	0.01449 (17)	0.02295 (18)	0.01437 (17)	0.00462 (13)	0.00081 (13)	0.00423 (13)
O1	0.0162 (18)	0.0241 (18)	0.0176 (17)	0.0048 (14)	0.0059 (14)	0.0023 (14)
N1	0.044 (3)	0.027 (2)	0.032 (3)	0.009 (2)	0.025 (2)	−0.001 (2)
C1	0.021 (3)	0.013 (2)	0.016 (2)	−0.0025 (19)	0.001 (2)	−0.0023 (18)
C2	0.045 (4)	0.016 (2)	0.024 (3)	0.010 (2)	0.018 (3)	0.003 (2)
C3	0.025 (3)	0.026 (3)	0.016 (2)	0.014 (2)	0.011 (2)	0.008 (2)
Sn2	0.01234 (17)	0.01365 (16)	0.01193 (16)	0.00425 (12)	0.00339 (13)	0.00231 (12)
I4	0.01251 (17)	0.01815 (17)	0.02403 (18)	0.00266 (13)	0.00420 (13)	0.00230 (13)
I5	0.01529 (18)	0.02042 (17)	0.02122 (18)	0.00887 (13)	0.00530 (13)	0.00606 (13)
I6	0.01956 (18)	0.02811 (19)	0.01277 (16)	0.00340 (14)	0.00347 (13)	0.00657 (13)
O2	0.028 (2)	0.0208 (18)	0.0155 (17)	0.0060 (15)	0.0108 (15)	0.0032 (14)
N2	0.035 (3)	0.030 (3)	0.022 (2)	0.005 (2)	0.015 (2)	0.003 (2)
C4	0.022 (3)	0.013 (2)	0.013 (2)	0.0072 (19)	0.0009 (19)	0.0001 (17)
C5	0.022 (3)	0.021 (2)	0.016 (2)	0.002 (2)	0.004 (2)	−0.0031 (19)
C6	0.014 (2)	0.029 (3)	0.016 (2)	0.009 (2)	0.0047 (19)	0.006 (2)

### Geometric parameters (Å, °)

**Table:**

Sn1—C1	2.146 (5)	Sn2—C4	2.147 (5)
Sn1—O1	2.347 (3)	Sn2—O2	2.330 (3)
Sn1—I1	2.6953 (4)	Sn2—I4	2.6987 (4)
Sn1—I2	2.7796 (4)	Sn2—I5	2.6880 (4)
Sn1—I3	2.6904 (4)	Sn2—I6	2.8060 (4)
O1—C3	1.244 (6)	O2—C6	1.262 (6)
N1—C3	1.324 (7)	N2—C6	1.313 (6)
N1—H1N	0.8800	N2—H3N	0.8800
N1—H2N	0.8800	N2—H4N	0.8800
C1—C2	1.521 (7)	C4—C5	1.526 (7)
C1—H1A	0.9900	C4—H4A	0.9900
C1—H1B	0.9900	C4—H4B	0.9900
C2—C3	1.512 (8)	C5—C6	1.506 (7)
C2—H2A	0.9900	C5—H5A	0.9900
C2—H2B	0.9900	C5—H5B	0.9900
			
C1—Sn1—O1	76.52 (16)	C4—Sn2—O2	77.38 (16)
C1—Sn1—I3	125.92 (14)	C4—Sn2—I5	129.25 (14)
O1—Sn1—I3	87.71 (9)	O2—Sn2—I5	89.69 (9)
C1—Sn1—I1	122.64 (14)	C4—Sn2—I4	118.64 (14)
O1—Sn1—I1	83.18 (9)	O2—Sn2—I4	84.58 (9)
I3—Sn1—I1	105.788 (15)	I5—Sn2—I4	108.400 (14)
C1—Sn1—I2	98.50 (14)	C4—Sn2—I6	96.42 (13)
O1—Sn1—I2	173.92 (9)	O2—Sn2—I6	172.63 (9)
I3—Sn1—I2	98.106 (14)	I5—Sn2—I6	97.373 (13)
I1—Sn1—I2	96.853 (14)	I4—Sn2—I6	95.120 (13)
C3—O1—Sn1	112.5 (3)	C6—O2—Sn2	111.5 (3)
C3—N1—H1N	109.5	C6—N2—H3N	109.5
C3—N1—H2N	109.5	C6—N2—H4N	109.5
H1N—N1—H2N	109.5	H3N—N2—H4N	109.5
C2—C1—Sn1	111.0 (4)	C5—C4—Sn2	110.1 (3)
C2—C1—H1A	109.4	C5—C4—H4A	109.6
Sn1—C1—H1A	109.4	Sn2—C4—H4A	109.6
C2—C1—H1B	109.4	C5—C4—H4B	109.6
Sn1—C1—H1B	109.4	Sn2—C4—H4B	109.6
H1A—C1—H1B	108.0	H4A—C4—H4B	108.1
C3—C2—C1	111.7 (4)	C6—C5—C4	111.5 (4)
C3—C2—H2A	109.3	C6—C5—H5A	109.3
C1—C2—H2A	109.3	C4—C5—H5A	109.3
C3—C2—H2B	109.3	C6—C5—H5B	109.3
C1—C2—H2B	109.3	C4—C5—H5B	109.3
H2A—C2—H2B	107.9	H5A—C5—H5B	108.0
O1—C3—N1	121.5 (5)	O2—C6—N2	121.2 (5)
O1—C3—C2	120.3 (4)	O2—C6—C5	120.6 (4)
N1—C3—C2	118.2 (5)	N2—C6—C5	118.2 (5)
			
C1—Sn1—O1—C3	6.4 (4)	C4—Sn2—O2—C6	5.8 (3)
I3—Sn1—O1—C3	−121.4 (3)	I5—Sn2—O2—C6	−124.7 (3)
I1—Sn1—O1—C3	132.4 (3)	I4—Sn2—O2—C6	126.8 (3)
O1—Sn1—C1—C2	−19.9 (3)	O2—Sn2—C4—C5	−20.4 (3)
I3—Sn1—C1—C2	57.1 (4)	I5—Sn2—C4—C5	58.6 (4)
I1—Sn1—C1—C2	−92.5 (4)	I4—Sn2—C4—C5	−97.0 (3)
I2—Sn1—C1—C2	163.6 (3)	I6—Sn2—C4—C5	163.6 (3)
Sn1—C1—C2—C3	31.0 (6)	Sn2—C4—C5—C6	32.4 (5)
Sn1—O1—C3—N1	−172.4 (4)	Sn2—O2—C6—N2	−169.0 (4)
Sn1—O1—C3—C2	9.8 (6)	Sn2—O2—C6—C5	11.7 (6)
C1—C2—C3—O1	−27.7 (7)	C4—C5—C6—O2	−30.2 (7)
C1—C2—C3—N1	154.4 (5)	C4—C5—C6—N2	150.5 (5)

### Hydrogen-bond geometry (Å, °)

**Table:**

*D*—H···*A*	*D*—H	H···*A*	*D*···*A*	*D*—H···*A*
N1—H1n···O2	0.88	2.29	3.085 (7)	150
N2—H3n···O1	0.88	2.26	3.018 (7)	145
N2—H4n···I1	0.88	3.06	3.784 (6)	141
